# Toward Practical Integration of Omic and Imaging Data in Co-Clinical Trials

**DOI:** 10.3390/tomography9020066

**Published:** 2023-04-10

**Authors:** Emel Alkim, Heidi Dowst, Julie DiCarlo, Lacey E. Dobrolecki, Anadulce Hernández-Herrera, David A. Hormuth, Yuxing Liao, Apollo McOwiti, Robia Pautler, Mothaffar Rimawi, Ashley Roark, Ramakrishnan Rajaram Srinivasan, Jack Virostko, Bing Zhang, Fei Zheng, Daniel L. Rubin, Thomas E. Yankeelov, Michael T. Lewis

**Affiliations:** 1 Department of Biomedical Data Science, Stanford University School of Medicine, Stanford, CA 94305, USA; 2 Dan L. Duncan Cancer Center, Baylor College of Medicine, Houston, TX 77030, USA; 3 Oden Institute for Computational Engineering and Sciences, Austin, TX 78712, USA; 4 Livestrong Cancer Institutes, Austin, TX 78712, USA; 5 Lester and Sue Smith Breast Center, Baylor College of Medicine, Houston, TX 77030, USA; 6 Department of Molecular and Cellular Biology and Radiology, Baylor College of Medicine, Houston, TX 77030, USA; 7 Department of Physiology, Baylor College of Medicine, Houston, TX 77030, USA; 8 Department of Medicine, Baylor College of Medicine, Houston, TX 77030, USA; 9 Department of Oncology, The University of Texas at Austin, Austin, TX 78712, USA; 10 Department of Diagnostic Medicine, The University of Texas at Austin, Austin, TX 78712, USA; 11 Department of Radiology, Stanford University School of Medicine, Stanford, CA 94305, USA; 12 Department of Medicine, Stanford University School of Medicine, Stanford, CA 94305, USA; 13 Department of Biomedical Engineering, The University of Texas at Austin, Austin, TX 78712, USA; 14 Department of Imaging Physics, The University of Texas MD Anderson Cancer Center, Houston, TX 77030, USA

**Keywords:** magnetic resonance imaging (MRI), multi-omics, breast cancer, cancer informatics, cancer modeling, radiomics

## Abstract

Co-clinical trials are the concurrent or sequential evaluation of therapeutics in both patients clinically and patient-derived xenografts (PDX) pre-clinically, in a manner designed to match the pharmacokinetics and pharmacodynamics of the agent(s) used. The primary goal is to determine the degree to which PDX cohort responses recapitulate patient cohort responses at the phenotypic and molecular levels, such that pre-clinical and clinical trials can inform one another. A major issue is how to manage, integrate, and analyze the abundance of data generated across both spatial and temporal scales, as well as across species. To address this issue, we are developing MIRACCL (molecular and imaging response analysis of co-clinical trials), a web-based analytical tool. For prototyping, we simulated data for a co-clinical trial in “triple-negative” breast cancer (TNBC) by pairing pre- (T0) and on-treatment (T1) magnetic resonance imaging (MRI) from the I-SPY2 trial, as well as PDX-based T0 and T1 MRI. Baseline (T0) and on-treatment (T1) RNA expression data were also simulated for TNBC and PDX. Image features derived from both datasets were cross-referenced to omic data to evaluate MIRACCL functionality for correlating and displaying MRI-based changes in tumor size, vascularity, and cellularity with changes in mRNA expression as a function of treatment.

## 1. Introduction

PDX models of cancer have proven to recapitulate much of the biology (mutations, copy number alterations, transcriptomic, and proteomic gene expression) and treatment responses of the matched tumors-of-origin to a high degree [1,2,3,4]. As such, the concept of co-clinical trials has emerged in which a cohort of human patients in a clinical trial is compared with a cohort of patient-matched (rarely), or more commonly, biosimilar PDX models in a pre-clinical trial, in which PDX-bearing mice are treated in a manner that mimics the clinical pharmacokinetic and pharmacodynamic features of the agent(s) used as closely as possible, and patient tumors and PDX are analyzed in an equivalent manner at both the phenotypic and molecular levels. A primary goal is to determine the degree to which PDX cohort responses recapitulate patient cohort responses at the phenotypic and molecular levels, such that pre-clinical and clinical trials can inform one another. If so, co-clinical trials offer the possibility that pre-clinical PDX-based studies can ultimately be used to develop predictive biomarkers of response, and identify mechanisms of resistance, in a safe, cost-effective manner, with clinical validation in subsequent trials.

Given the biological and phenotypic consistency between PDXs and their respective tumors-of-origin from patients, we hypothesized that features obtained from an analysis of pre-, on-, or post-treatment imaging (e.g., magnetic resonance imaging (MRI), positron emission tomography (PET), X-ray computed tomography (CT), ultrasound) or histology/pathology can be correlated closely with pre-, on-, and post-treatment changes in omic features to yield meaningful, and potentially actionable, results. 

### 1.1. Central Challenges in Co-Clinical Trials

The central challenges in co-clinical trials are at least fourfold. First, what is required to establish the relevance of the PDX cohort being used such that the clinical and preclinical data are as comparable as possible, and can thus inform one another? Second, how is the large amount of imaging and omic data generated effectively managed? Third, how should the imaging and omic data be simultaneously, systematically, and easily interrogated? Fourth and finally, how should the commonalities and differences between datasets be evaluated both qualitatively and quantitatively, and subsequently visualized?.

### 1.2. “Credentialling” of PDX Models Is Essential for Co-Clinical Trials to Be Translationally Relevant

In order for co-clinical trials to be translationally relevant, the PDX preclinical cohort must reflect as much biology as possible relative to the clinical patient cohort. Accordingly, work from many groups, including investigators associated with large consortia, such as the PDXNet program of the U.S. National Cancer Institute (www.pdxnetwork.org) [5,6] and the EuroPDX consortium (www.europdx.eu) [7,8], have paved the way for validating the robustness with which human-in-mouse PDX biology recapitulates the biology observed in human patients. These groups have conducted detailed analyses of genomic copy number alterations and deleterious gene variants using whole exome and, in some cases, whole genome DNA sequencing (WES and WGS, respectively), corroborated by array-based comparative genomic hybridization methods, all of which compare well with the tumors-of-origin, and with samples in The Cancer Genome Atlas (TCGA) [1,4,6,9,10,11,12]. Furthermore, transcriptomic (RNAseq and microarray-based) and mass spectrometry-(MS-) and reverse phase proteomics (RPPA) gene expression profiles correlate well with those of patient-matched tissue [13].

### 1.3. Data Challenges and the Conception of MIRACCL

Managing large amounts of data is inherently difficult for any clinical or pre-clinical trial, particularly when imaging and omic analyses are part of the study design. The problem is amplified if multiple omic platforms are employed. With respect to how these data are interrogated, current tools only allow one study to be interrogated at a time, with integration between clinical and preclinical analyses performed as a secondary activity. Similarly with visualization, separate clinical and preclinical analyses are typically visualized separately and integrated only when a figure is constructed.

### 1.4. Conceptualization of MIRACCL

To address these challenges, we have developed a conceptual framework and workflows to guide in the conduct of co-clinical trials, as well as a web-based visualization and analysis tool that provides non-computational biologists and clinicians a tool for analyzing co-clinical trial data that is intuitive, easy to use, and analytically robust. We have named this tool MIRACCL (molecular and imaging response analysis of co-clinical trials, https://miraccl.research.bcm.edu/). Currently, the tool allows for comparison of changes in pre- (Timepoint 0, T0) vs. on-treatment (Timepoint 1, T1) tumor longest diameter, tumor volume, MRI-based measures of vascularity (dynamic contrast-enhanced magnetic resonance imaging, DCE-MRI) and cellularity (diffusion weighted magnetic resonance imaging, DW-MRI). MIRACCL correlates these image feature changes with changes in mRNA expression as a function of therapy response at the end-of-treatment. In future releases, pre- vs. post-treatment (Timepoint 2, T2) image feature comparisons will be enabled, and analytes will be extended to include DNA (WES for deleterious variants and copy number alterations), as well as MS-based protein expression, with additional analytical tools for their analysis. While current prototyping efforts for MIRACCL are geared exclusively toward an integrated analysis of co-clinical trials, the tool already has the capability to analyze and display stand-alone clinical or pre-clinical cohort datasets, independent of its co-clinical data management, analysis and display capabilities, and can thus be used for any study in which integrated imaging and omic analyses are desired.

**Figure 1:**
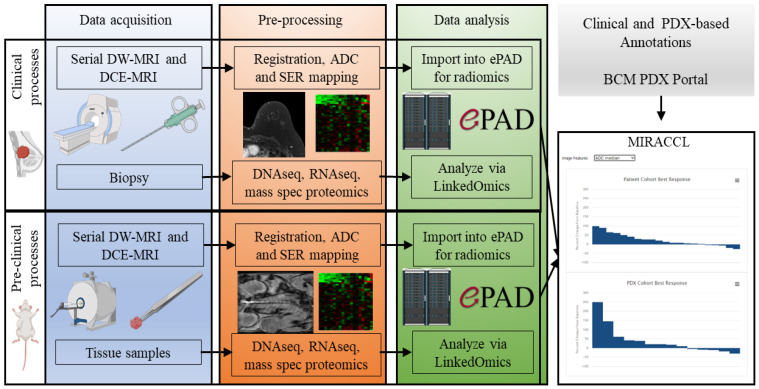
An illustrative overview of the flow of data and analysis within MIRACCL. The three columns describe the major components of data acquisition (blue), pre-processing (orange), and data analysis (green). The rows that cut across the three columns correspond to the clinical (top row) and pre-clinical settings (bottom row). Both DW-MRI and DCE-MRI are obtained for both settings, as are tissue samples. In the pre-processing stage, images are converted into quantitative parameter maps corresponding to the apparent diffusion coefficient (ADC, from DW-MRI) and the signal enhancement ratio (SER, from the DCE-MRI). Additionally, during this stage, the DNAseq (WES), RNAseq, and mass spectrometry proteomics are performed. The resulting data and images are then delivered to LinkedOmics and ePAD to summarize the output for subsequent input into MIRACCL.

MIRACCL represents the integration of three existing web-based platforms (Figure 1). The first is the Baylor College of Medicine PDX Portal (https://pdxportal.research.bcm.edu/pdxportal), in which clinical and PDX-related data are stored, managed, and displayed. The second is ePAD (electronic physician annotation device) (http://epad.stanford.edu) [14], in which images and associated image features are stored, organized, analyzed, and results are displayed. The third is LinkedOmics [15], in which cohort-based multi-omic and other quantitative metrics (e.g., treatment responses and selected quantitative image features), can be stored, analyzed, and displayed. Rather than re-inventing the functionality of these three platforms, MIRACCL integrates their functionalities in a user-friendly, intuitive, web-based user interface that leverages the wealth of PDX-related and clinical data, the robust informatics capabilities of LinkedOmics, and the high-quality displays enabled in ePAD, thus obviating the need to involve multiple specialists (e.g., PDX developers, statisticians, bioinformaticists, database architects, programmers). The goal is to allow end users to analyze and display their data without the need for access to a wide variety of experts and separate computational tools.

### 1.5. The Original Impetus for Developing MIRACCL

**Figure 2:**
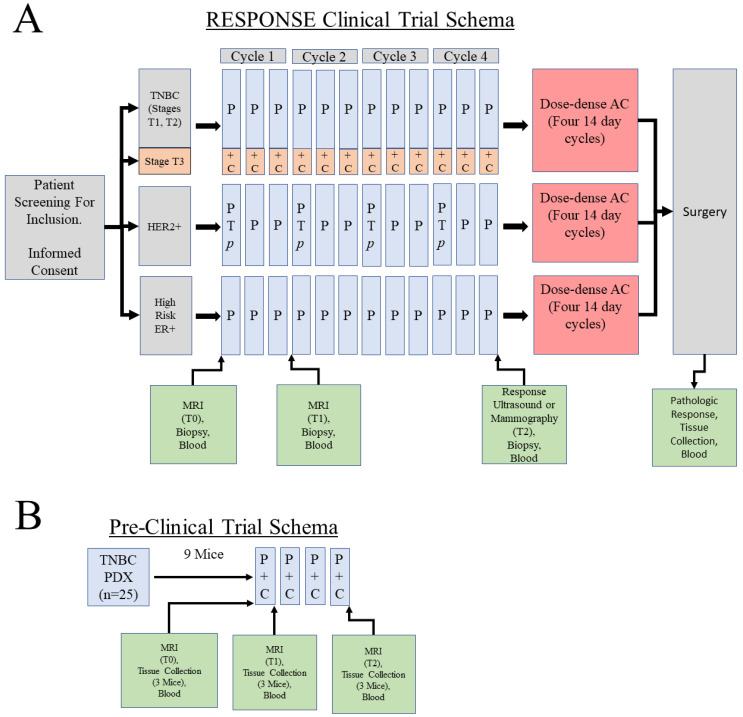
Co-clinical trial design. (**A**) Overall RESPONSE clinical trial schema. For the purposes of this study, only the Stage T3 TNBC portion of the trial will be used in which patients will receive weekly paclitaxel (P) for a period of 12 weeks (four 21-day cycles) in combination with carboplatin (+/− pembrolizumab, *p*) given on day 1 of each of the four cycles. Imaging will be conducted at three timepoints. The first two timepoints will use MRI, while the third timepoint will use either ultrasound or mammography to assess response to the paclitaxel-containing regimens. Only the baseline biopsy is required in the trial; all others are optional. Our goal is to recruit 50 TNBC patients who will agree to have the first two biopsies for the generation of omic data (WES, RNAseq, MS-Proteomics). HER2+ patients will receive paclitaxel in combination with trastuzumab (T) and pertuzumab (P), while high risk ER+ patients will receive paclitaxel as a single agent. (**B**) PDX-based preclinical trial schema. Mice will receive four weekly cycles of combination paclitaxel/carboplatin with three MRI timepoints.

The original impetus for developing MIRACCL was to support a project in which we ask the question whether changes in image features, as well as changes in gene expression after one cycle of treatment (on-treatment), can predict ultimate treatment response at end-of-treatment (in our case, four cycles of combination carboplatin/paclitaxel (CP) prior to subsequent anthracycline/cyclophosphamide (AC) treatment) in a co-clinical trial using both a pre-clinical cohort of TNBC breast cancer PDX-bearing mice, and a breast cancer cohort consisting of the high grade TNBC arm of the upcoming “all comers” RESPONSE clinical trial (please see ClinicalTrials.gov Identifier: NCT05020860). In this arm, stage T3 “triple-negative” (TNBC) tumors would be treated with combination carboplatin/paclitaxel followed by anthracycline/cyclophosphamide (AC), with pre-, on-, and post-treatment imaging using MRI (pre- and on-treatment) and ultrasound (post paclitaxel). Stage T1 and T2 TNBC would be treated with paclitaxel only followed by AC, and are not considered in our prototyping efforts. Likewise, tumors expressing estrogen receptor (ER) (+/− progesterone receptor (PR)), or showing amplification/overexpression of ERBB (HER2+) will receive the standard of care therapies, and are not considered in our prototyping effort. An overview of this trial is shown in Figure 2A. The PDX-based co-clinical trial schema is shown in Figure 2B. If successful, it may be possible to spare patients ineffective treatment(s) and allow physicians to change treatments more rapidly to a potentially more effective one.

## 2. Approach and Methods

### 2.1. Clinical Cohort Selection for Generation of Data for MIRRACL Prototyping

In preparation to host these data, in this report, we evaluate the functionality of a first generation MIRACCL prototype using simulated co-clinical trial imaging and omic datasets. The simulation employs the recently released MRI data of paclitaxel-treated, triple-negative breast cancer from the I-SPY2 clinical trial (Investigation of Serial studies to Predict Your Therapeutic Response with Imaging and Molecular Analysis 2) (NCT01042379) (https://www.ispytrials.org/) (Supplementary Figure S1) in which MRI data were taken at multiple timepoints, including pre-treatment (T0), after the first cycle of paclitaxel (T1, on-treatment) and after completion of the paclitaxel treatment (T2, post paclitaxel treatment +/− experimental agent for TNBC). All patients in the I-SPY2 trial go on to receive combination adriamycin/cyclophosphamide (AC), with an additional MRI, prior to surgery to remove the primary tumor. These AC data and associated images were not used for our prototyping purposes. With respect to omics, gene expression at the mRNA level was only evaluated at baseline (T0, pre-treatment). Thus, T1 mRNA expression was simulated by a random permutation of T0 expression values.

For prototyping purposes, a selected subset of TNBC data from the I-SPY2 trial MRI data were used in conjunction with our own pre-clinical, PDX-based, MRI data evaluating response of PDX models of triple-negative breast cancer to treatment with combination CP. In both instances, pre- (T0), on- (T1, after the first weekly cycle of a four week paclitaxel only treatment regimen), and post-treatment (T2, after four weekly cycles of paclitaxel) images were captured. 

Vascular and cellularity features from the MRI data were derived for both the I-SPY2 clinical trial images (T0 vs. T1) and the PDX cohorts (T0 vs. T1) for cross-referencing between clinical and pre-clinical studies in the MIRACCL tool as a function of post-treatment response (T0 vs. T2 changes in tumor volume) (note that only T0 vs. T1 comparisons are currently implemented on MIRACCL). We also generated simulated paired T0 and T1 omic data at the mRNA expression level for initial evaluation purposes by a random permutation of pre-treatment mRNA expression tables. The ultimate goal is to expand the image and omic data types to allow correlation relative to the quantitative assessment of treatment responses at multiple timepoints.

### 2.2. PDX Selection and Chemotherapy Treatment

At BCM, we have generated 88 PDX models of human breast cancer, 54 of which represent TNBC. Additional PDX models were imported to BCM from other PDX development sites, including the Huntsman Cancer Institute (Dr. Alana Welm), Washington University (Dr. Shunquian Li), the University of Michigan (Dr. Max Wicha), and the Mayo Clinic (Dr. Matthew Goetz). Additional models were contributed by Xentech Inc. The combined collection consists of 261 breast PDX models available for use in co-clinical trials, including the 173 PDX models of TNBC, a subset of which we employed for the present study [3,12,13,16,17]. Annotations describing the PDX models used in this study can be found in the BCM PDX portal (https://pdxportal.research.bcm.edu/) where PDX genomics data, patient clinical annotations, and contact information for using BCM’s PDX resource is available in an open access format.

All animal experiments were conducted with approval from the BCM Institutional Animal Care and Use Committee. The PDXs chosen for combination carboplatin/paclitaxel treatment and MRI were selected from among 50 TNBC PDX previously determined to show either resistance or sensitivity to combination carboplatin and docetaxel treatment [1], as well as from new TNBC models of interest. 

Fresh PDX tumor tissue fragments (approximately 1 mm^3^) were transplanted into the epithelium-free “cleared” #4 fat-pad (right inguinal) of four-week-old SCID/beige (Envigo, C.B-17/IcrHsd-Prkdc^scid^Lyst^bg-J^) mice as described previously (Ref. [18] modified from Ref. [19]). When tumors reached an average size of ~175 mm^3^ (length × width^2^ × 0.5), animals were randomized (*n* = 3/group) into pre-treatment (T0), on-treatment (T1, after one cycle) or post-treatment (T2, after four-weeks) treatment arms. Pre-treatment MRI was performed on all nine mice, and three mice were sacrificed with tissue and blood collected to serve as T0 samples. The remaining six animals were treated intraperitoneally with 50 mg/kg carboplatin (740278, McKesson) and 33 mg/kg paclitaxel (T7402, Millipore Sigma, St. Louis, MO, USA) (Supplementary Table S1), each shown previously to represent human-equivalent doses based on PK/PD analyses conducted by the National Cancer Institute in service to PDXNet projects. Three of the six mice were sacrificed after one cycle to serve as T1 samples. The three remaining mice received three additional weekly treatments and were sacrificed at the four-week timepoint to serve as T2 samples. Portions of tissue from the brain, liver, and lungs were also collected for FFPE blocks, and the remaining tissue was minced to a uniform mixture and snapped frozen. Whole lymph nodes and ovaries were also collected for FFPE and snap frozen specimens. 

Strictly for prototyping purposes, each PDX-bearing mouse was treated as a unique “patient” so that the cohort size did not appear too small relative to the selected subset of the I-SPY2 image collection used.

### 2.3. PDX MRI Acquisition and Analysis

#### 2.3.1. Diffusion Weighted MRI (DW-MRI)

The microscopic, thermally induced behavior of molecules randomly moving is known as self-diffusion or the Brownian motion. The rate of diffusion in cellular tissues can be described by the apparent diffusion coefficient (ADC) that depends on the number and separation of barriers encountered by a diffusing water molecule. DW-MRI is an MRI technique designed to map the ADC. We used a diffusion-weighted spin echo approach with *TR*/*TE* = 2600 ms/31 ms with eight acquisition and *b*-values of 0, 150, and 700 s/mm^2^ (with δ/Δ = 3 ms/15 ms), an acquisition matrix of 128 × 128 × 16 with a field of view of 35 mm × 40 mm × 15.6 mm field-of-view, yielding a voxel resolution of 273 μm × 312.5 μm × 97.50 μm.

#### 2.3.2. Dynamic Contrast-Enhanced MRI (DCE-MRI)

DCE-MRI refers to the serial acquisition of heavily *T*_1_-weighted images before and after the injection of a paramagnetic contrast agent. Each image corresponds to one time point, and each voxel in each image set (or a whole region of interest) gives rise to its own time course that can be analyzed qualitatively to report various features of the enhancement pattern, or quantitatively with a pharmacokinetic model to estimate physiological parameters, such as the contrast agent volume transfer constant (*K^trans^*, related to vessel perfusion and permeability), the extravascular extracellular volume fraction (*v_e_*), the efflux rate constant (*k_ep_* = *K^trans^*/*v_e_*), and the plasma volume (*v_p_*). In this proof-of-concept study, we make use of a semi-quantitative analysis of the DCE-MRI data to return the signal enhancement ratio (SER).

For our studies, we used a 9.4 T Bruker (Billerica, MA, USA) Avance NEO MRI with Paravision 360 software. Prior to imaging, an MRI-compatible catheter was placed in the tail vein for delivery of the contrast agent. Mice were induced with 5% isoflurane and transferred supine to the animal-imaging holder, and then transferred to the imaging instrument where 2% isoflurane was administered continuously via nose cone. A pressure-sensitive pillow was placed on the abdomen to monitor respiration. Temperature was monitored using a rectal probe and maintained at 37 °C using a heating system. A pre-contrast *T*_1_ map was acquired via an inversion-recovery snapshot acquisition with repetition time = 12 s, inversion time = 0.250–11 s, and number of excitations = 2. A three-dimensional spoiled gradient recalled echo sequence with a fixed flip angle of 16 degrees over 150 repetitions was used to obtain the dynamic data. For the spoiled gradient recalled echo sequence, the repetition time = 6 ms, echo time = 1.93 ms, and an acquisition matrix = 128 × 128 × 16 over a 3.0 cm × 3.0 cm × 2.5 cm field of view, yielding a voxel resolution of 0.234 mm × 0.234 mm × 1.562 mm. Two minutes into the dynamic acquisition, 0.1 mmol/kg of Magnevist was injected via the tail vein catheter.

#### 2.3.3. Image Analysis and Feature Determination

The pre-clinical MRI data were loaded into MATLAB (R2021a, Mathworks, Nattick, MA, USA) for image processing to derive parameter maps (signal enhancement ratio (*SER*) and *ADC*) and tumor segmentations. Tumor regions of interest were manually drawn on the difference between the pre- and post-contrast images at each visit. The *SER* was then calculated using Equation (1):

(1) $$SER=\frac{S_{early}-S_{baseline}}{S_{late}-S_{baseline}},$$

where *S_baseline_* is the average of the first 10 time points, *S_early_* is the peak signal intensity observed in the tumor ROI, and *S_late_* is the signal intensity 6 min after the peak as observed. 

The *ADC* maps were obtained using standard approaches by fitting Equation (2) to the DW-MRI data:

(2) $$S\left(b\right)=S_{0}e^{-ADCb},$$

where *S*(*b*) is the signal intensity acquired with diffusion weighting *b*, and *S*_0_ is the signal intensity in the absence of diffusion gradients. *ADC* values were estimated on a voxel-by-voxel basis using the *lsqcurvefit* function in MATLAB. *ADC*, *SER*, and the accompanying segmentation masks were scaled for display in ePAD and saved as DICOM segmentation objects. These DICOM segmentation objects were loaded into ePAD for calculation of the longest diameter of the tumor, tumor volume, mean/median *SER*, and mean/median *ADC*. 

### 2.4. Human Breast Cancer Image Selection and Analysis

The I-SPY2 trial was designed to evaluate paclitaxel combined with an experimental agent relative to a single agent paclitaxel (control). While not strictly “co-clinical” with our PDX study due to being a single agent therapy vs. the combination used in the PDX-bearing mice, these clinical MRI data are well-suited for prototyping MIRACCL.

Human MRI data were acquired retrospectively from the I-SPY 2 trial. More specifically, clinical images consisted of a subset of 573 breast MRIs from 191 patients in I-SPY 2 that were used for the breast multiparametric MRI for the prediction of neoadjuvant treatment response. For prototyping purposes, a selection of 21 TNBC patients were used who received single agent paclitaxel. This dataset included MRI performed at three timepoints that are relevant to this study: (1) pre-treatment (T0), (2) on-treatment (T1) after 3 weeks of paclitaxel (with or without an experimental agent), and (3) “post”-treatment (T2) after completion of 12 weekly cycles of paclitaxel, but before subsequent treatment with four cycles of AC and another MRI at the end of treatment prior to surgery that is not relevant to our prototyping effort. 

MRI was performed on 1.5 T or 3.0 T scanners at one of 10 study sites, and consisted of: a localization scan, *T*_2_-weighted sequence, diffusion weighted imaging (DWI) sequence (*b* = 0, 100, 600, 800 s/mm^2^, 3-directions), and *T*_1_-weighted dynamic contrast enhanced (DCE) sequence (temporal resolution between 80 and 100 sec acquired for at least 8 min post-injection of contrast agent). Tumors were identified from post-contrast DCE subtraction images and manually segmented by selecting regions with hyperintensity at the high b-value image while avoiding adipose and fibroglandular tissue, biopsy clip artifacts, and regions of high T2 signal (e.g., seroma and necrosis), as previously described [20].

Segmentations of the I-SPY2 data were loaded into MATLAB for image processing to yield SER and ADC maps as described above. Non-PHI (protected health information) identifiers and MRI scan parameters were parsed from DICOM headers of each scan. DW-MRI and DCE-MRI data, along with the tumor segmentations and derived SER and ADC maps were scaled for display in ePAD and saved as DICOM segmentation objects. These DICOM segmentation objects were loaded into ePAD for calculation of the longest diameter of the tumor, tumor volume, mean/median SER, and mean/median ADC.

### 2.5. Image and Feature Curation and Display in the ePAD System

ePAD is an open-source platform that enables image viewing, annotation, and analysis of cohorts of patient and animal images [14]. It supports the DICOM standard format for image data. ePAD saves all image annotations in the annotation and image markup (AIM) format [21], a standard that enables interoperability, and that has been harmonized with DICOM. ePAD runs as a virtual machine and is accessible via a web browser (the URL to access ePAD is specific to the institution that deploys it). ePAD consists of three main components: (1) a web-based radiology workstation for viewing images and annotating them using templates that contain common data elements with controlled terms useful for image labeling for analysis, (2) a database for managing images and regions of interest on the images that circumscribe cancer lesions, and (3) a web services API, which allows invoking ePAD from web applications.

### 2.6. Simulation of Pre- and On-Treatment Human and PDX RNAseq Datasets by Random Permutation of Baseline mRNA Expression

For the clinical gene expression dataset, gene level batch-corrected and normalized RNA expression data from the I-SPY2 trial (GSE194040) representing TNBC patients were used as pre-treatment (T0) gene expression data for the prototype patient dataset. These RNAseq data were patient matched with corresponding T0 MRI data. The I-SPY2 expression dataset contains 19,134 genes for 988 patient samples, which were then subset to 18,139 gene symbols that overlapped with the genes present in the PDX RNAseq dataset. To simulate T1 gene expression data, the T0 expression tables were randomly permuted by multiplying each data point (i.e., list of normalized counts) with one of 4 numbers between 0.5 and 1.5, where the vector of 4 numbers was picked at random but fixed per patient. 

For the pre-clinical study, because the PDX cohort was considerably smaller than the I-SPY2 cohort, each mouse imaged above was treated as an individual “patient” to increase our sample size despite the fact that, for each PDX treated and imaged, there were three biological replicates. One mouse from each set of three retained the actual PDX identifier and associated image and pre-treatment RNAseq data. For the remaining two mice in each set, we replaced the original PDX identifier with an identifier for a different PDX that responded to treatment in a similar manner as the imaged PDX. We also used the baseline T0 RNAseq data for the replacement PDX. In doing this, we attempted to maximize the probability that genes identified in the I-SPY2 dataset as correlating with treatment response would have overlapped with the genes identified in the PDX dataset.

Raw RSEM counts obtained through our RNAseq pipeline (Xenome -> RSEM + STAR) were filtered for selection of expression data corresponding to this subset of PDX models and used as pre-treatment RNAseq data. 

As with the I-SPY2 mRNA expression data, to simulate on-treatment (T1) RNAseq data for PDX, the chosen pre-treatment RNAseq counts were also randomly permuted by multiplying each data point (i.e., list of raw counts) with one of 4 numbers between 0.5 and 1.5, where the vector of 4 numbers was picked at random but fixed per PDX model. This resulted in generating a new set of raw counts that correlated with each original set of raw counts at a coefficient 0.5 ≤ r^2^ ≤ 0.8. This meant that the simulated raw counts correlated moderately well with their original data points. Code is available in Supplementary Materials. 

### 2.7. Omic Data Management and Storage via Inclusion into LinkedOmics

LinkedOmics is a cohort-based analysis tool that performs on-demand association analysis within and across cohorts in a study using multiple omics and phenotypic datatypes [15]. It computes and ranks genes associated with a user-selected feature in a cohort and visualizes the results in tables and statistical plots, such as volcano plot and heatmap. The completed jobs are stored for the user and can be compared across cohorts. For this project, both simulated gene expression data and quantitative image feature data were stored in the database of LinkedOmics. LinkedOmics provides an association analysis between imaging and response data and omics data for integration with MIRACCL. An API was designed for communications between the two web applications to run the analysis, and to return results for display in MIRACCL.

## 3. Results

### 3.1. Overall Design of MIRRACL: A Web-Based Tool Enabling the Analysis and Display of Co-Clinical Trial Data

MIRACCL was designed to address the need for presenting and analyzing data generated across multiple domains; in this case, clinical, imaging, PDX laboratory, and bioinformatics in a single location. Adding to the complexity of comparing data across domains is the need to track data over time, as both patients and PDX progress through sampling and treatment events during a study. These multifactorial datasets must be integrated across technologies to enable interactive management and discovery.

**Figure 3:**
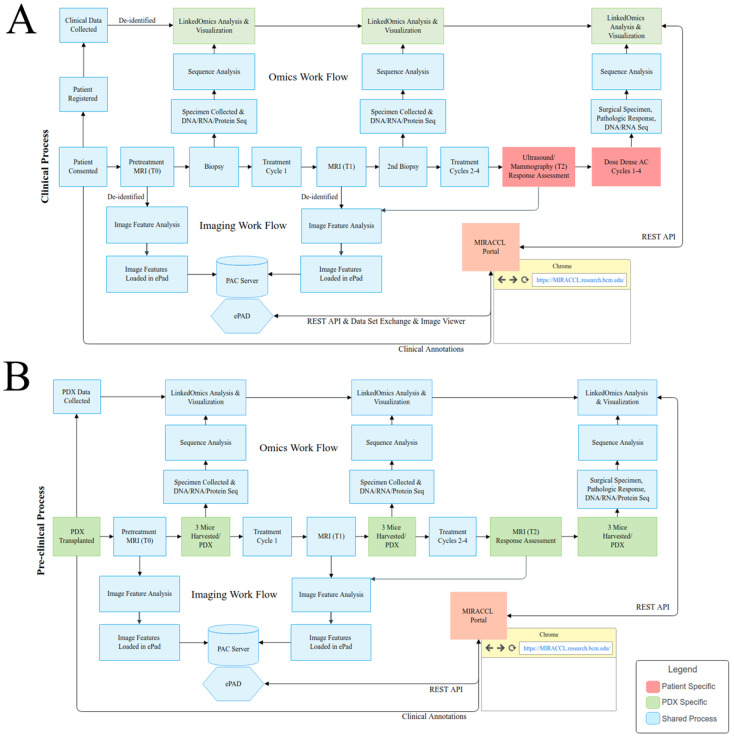
(**A**) Clinical workflow associated with a co-clinical trial. (**B**) Pre-clinical workflow associated with a co-clinical trial. The study workflows are diagramed centrally within each figure with specimen collection timepoints for omics generation shown above the study workflow and imaging collection timepoints shown below the workflow. Workflow steps shown in blue are standard across both the patient and PDX cohort while patient specific steps are shown in salmon and PDX specific steps in green.

Our first design task was to identify the requirements of the clinical and preclinical workflows; these requirements are depicted in Figure 3, which models the events and interactions for the patient clinically (Figure 3A) and for the pre-clinical or PDX-based studies (Figure 3B). Following the initiation of the diagram from left to right, high-level data collection timepoints and data generation events, along with the methods of interactions, are shown (sequencing data generation and handling on the top, and imaging generation and handling on the bottom). Figure 3B displays events in blue to indicate events in common across both the pre-clinical and clinical workflows, while events in green represent PDX-specific study events. While a majority of events are shared given the same study design between patient and PDX, patient consenting, clinical data collection, and select laboratory processes are unique to each cohort. Throughout, as either MRI takes place or specimen collection occurs, the data are collected and stored in the native format. For example, patient MRI data are stored in a PACS system, and biospecimens are sent out for multi-omic characterization, with results being returned to the bioinformatics group for post-processing. Rather than attempting to replace the functionality of any of these systems which are established for the storage and viewing of those specific data types, the portal integration service was constructed under the concept of leveraging the best available tools and presenting the integrated data in a manner which allows cross comparison between models and patients.

### 3.2. MIRACCL Architecture and Distributed Data Model

The architecture and data model of MIRACCL was designed to avoid generating multiple copies of very large genomics and imaging datasets. MIRACCL assumes the role of an integration portal providing multiple methods for comparison and tracking of PDX and patient subjects throughout the complex workflows of co-clinical studies. Thus, none of the DICOM files nor sequencing data has been loaded into MIRACCL. Rather, it works through a series of service interfaces to retrieve data from the native source upon request.

#### 3.2.1. Application Architecture

The MIRACCL user interface (UI) consists of multiple open-source plugins in combination with proxies and API to the external resources. The client-side utilizes HTML, CSS, ReactJS, JavaScript, Bootstrap, and jQuery for browser visibility and user inputs. The interface elements are broken down into the following categories:

1. Input Controls—JSF (Java Servlet Faces—Primefaces)
2. Navigation Components—Bootstrap
3. Informational Components—HTML, CSS
4. Interconnectivity with other applications—ReactJS, JavaScript, jQuery
5. Graphs and Datatables—Highcharts, JSF datatable

The server side uses Java, HTTP requests, and Ajax to react to user inputs, and return the appropriate content to display on the user interface. The middle layer consists of multiple web services and APIs that makes connection to ePAD and returns images to the MIRACCL user interface.

#### 3.2.2. Data Interaction with the BCM PDX Portal

Clinical and PDX-related meta-data are curated in a local relational database. For patients, these data include, but are not limited to, clinical diagnosis, age at diagnosis, race and ethnicity, as well as clinically relevant biomarker expression and mutations. The BCM PDX portal provides the source data for the PDX models currently displayed in MIRACCL. The local Oracle database schemas for the PDX portal and MIRACCL are linked through a one-directional database link which enables data in the BCM PDX portal to be visible to the MIRACCL schema. Thus, the underlying database used with the MIRRACL platform itself does not need to be duplicated, thereby obviating the need to update data in two locations.

Patient and PDX meta-data from external sources can also be directly inserted in the MIRACCL tables or read from the database link for shared local records without affecting the PDX portal. This data transfer method ensures that the data model is consistent in both portals while also providing external studies to be incorporated in the MIRACCL site without being present in the BCM PDX portal in the future.

#### 3.2.3. Data Interaction with ePAD

**Figure 4:**
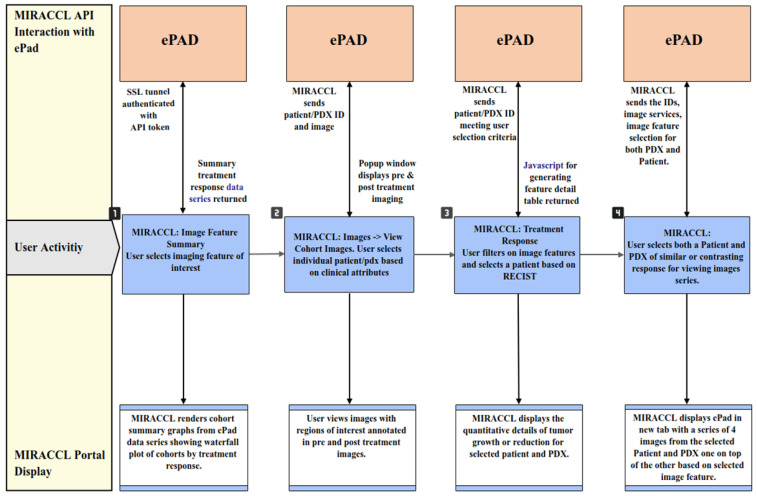
Interactions between ePAD and MIRACCL. User activity within MIRACCL in steps one through four. Imaging data analysis API requests from MIRACCL to ePAD are shown on the top row while resulting MIRACCL user interface (UI) responses are shown on the bottom row.

Imaging data are post-processed and loaded onto ePAD for image viewing and quantification of regions of interest. Limited meta-data and project information is stored in a local database server in a two-tiered web application and database approach. MIRACCL uses Hibernate SQL to retrieve data from this database. When the user selects a project of interest from MIRACCL, both the PDX and patient cohorts are retrieved from the database, along with demographics and image series identifiers for both groups. Within the Image Summary tab of MIRACCL, users can select the imaging feature of interest (e.g., longest diameter, volume, SER median and max, or ADC median and max). This selection, along with the cohort meta-data, are sent from the MIRACCL API to ePAD, as displayed in Figure 4 steps 1 and 2.

The ePAD API services then retrieve pre- and on-treatment response data tables and visualizations as seen in Figure 4, Steps 3 and 4. These two service APIs are secured and authorized via API tokens and encrypted URLs. The user interface does not consume these services directly, but via a proxy API service layer which is also composed of APIs in order to isolate the MIRACCL UI from changes to these independent applications.

#### 3.2.4. Interaction with LinkedOmics

LinkedOmics (described above in Section 2.7) is partially integrated into the MIRACCL portal. The integration of LinkedOmics allows for the correlation of gene expression values, or changes in gene expression values between two conditions (e.g., pre- vs. on-, or pre- vs. post-treatment), with quantitative imaging features, or their changes, as well as with clinical features. Navigation is achieved through a common MIRACCL query interface where users select the imaging or clinical feature of interest along with the study time point (pre-, on- or post-treatment). MIRACCL issues an API call to LinkedOmics, which returns genes with the most significant associations. MIRACCL renders the returned results in tables, and upon user demand, issues an API call to request the associated statistical plots. 

### 3.3. Data Display and Analyses in MIRACCL

#### 3.3.1. Evaluating Treatment Response

MIRACCL analyzes treatment response in human subjects and PDX animals by invoking ePAD’s response analysis tool that computes changes in a selected image feature between study timepoints. For prototyping purposes, we currently only consider pre- (T0) vs. on-treatment (T1) imaging feature changes. ePAD then generates a treatment response table listing the cancer lesion(s) in a human or animal subject, and the response based on comparison of a selected imaging feature (e.g., longest diameter, volume, SER median and max, or ADC median and max) between the T0 vs. T1 timepoints. Additional functionality will be added over time to allow additional comparisons (e.g., T0 vs. T1, T0 vs. T2, and T1 vs. T2).

The lesion name, lesion location, and lesion assessments on the baseline (T0) and first follow-up study (T1) are displayed, as well as the date and modality of the imaging studies. The cancer assessments include the imaging biomarker value of target lesions, and the response rate (RR) from the baseline (the change in the imaging biomarker value at a given treatment timepoint from the baseline value). In future versions of MIRACCL, response will be based on a comparison of the value of the imaging biomarker of the cancer lesion at the T0 vs. T2 timepoints, and will be given a clinically relevant treatment response category (e.g., residual cancer burden (RCB0-3), or a RECIST 1.1 category for patient tumors: progressive disease (PD), stable disease (SD), partial response (PR), or complete response (CR), or a modified RECIST (mRECIST) for PDX, as we published previously [13].

#### 3.3.2. Evaluating Treatment Response in Clinical and Pre-Clinical Cohorts

**Figure 5:**
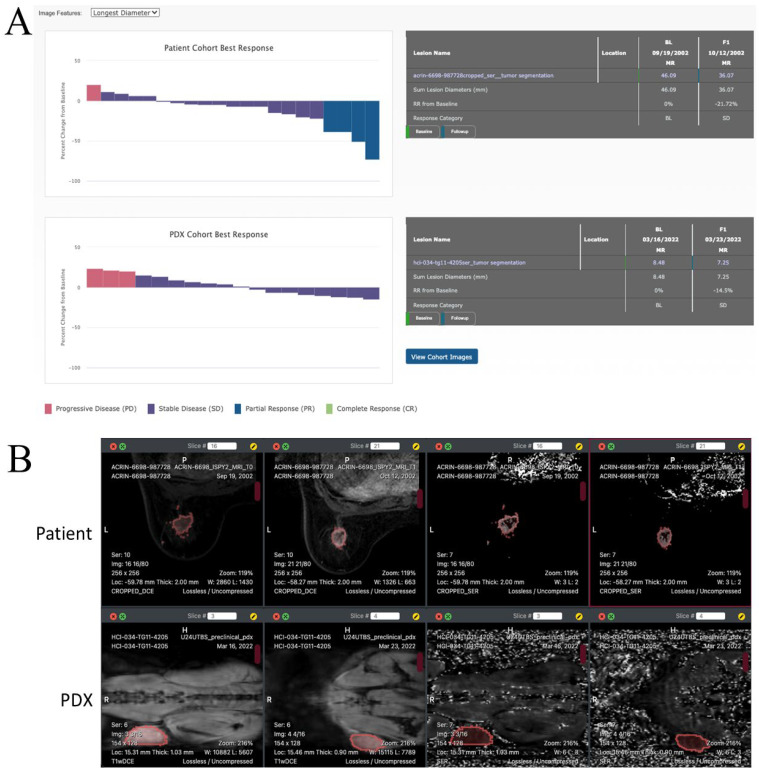
(**A**) Waterfall plot in MIRACCL of a treatment response in a cohort of patients (left) and in the corresponding cohort of PDX animals (right) based on the longest diameter of cancer lesions in pre-treatment and on-treatment images. These plots are generated by ePAD and displayed by MIRACCL. (**B**) Patient (top row) and corresponding PDX (bottom row) images as displayed in MIRACCL (tumor outlined in red). From left to right in the top row: T1W pre-treatment, T1W on-treatment, SER pre-treatment, and SER on-treatment for a patient from the I-SPY2 cohort. The bottom row shows four images of a biosimilar PDX model. From left to right: T1W pre-treatment, T1W on-treatment, SER pre-treatment, and SER on-treatment.

MIRACCL creates a waterfall plot to display the treatment response in cohorts of patients or PDX by invoking a procedure in ePAD to produce this plot. ePAD uses a selected imaging feature as the basis for assessing treatment response based on pre- and on-treatment images in each patient or PDX. Bars in the waterfall plot are ordered from the smallest to the greatest response. Figure 5A shows the waterfall plot created by MIRACCL for a cohort of human subjects and corresponding PDX using the longest diameter of a cancer lesion for assessing treatment response. Similar plots for tumor volume, SER and ADC values can also be generated (not shown).

The waterfall plots in the treatment response page of MIRACCL are interactive. If the user clicks on a bar in the plot, MIRACCL shows the response table for the patient corresponding to the bar in the plot (Figure 5A). The images and region of interest drawn on the images to circumscribe cancer lesions for that patient and corresponding PDX can be viewed by clicking on the View Cohort Images button after selecting the patient and PDX animal in the waterfall plots. This displays the images to enable user evaluation of the regions of interest evaluated in the patient and PDX (Figure 5B).

#### 3.3.3. Image Viewing

The ‘Cohort Images’ page of MIRACCL displays a list of all the patients in a co-clinical trial who had imaging studies (Supplementary Figure S2). The user can review images in a particular patient or PDX animal model by clicking on the ‘Pre- vs. On-Treatment Images’ link for that patient. MIRACCL then displays the pre- and on-treatment imaging studies for that patient or the associated PDX animal model using the ePAD image viewer that is integrated into MIRACCL. Additional functionality will be added to allow visualization of pre-, on-, and post-treatment images simultaneously.

#### 3.3.4. Omics Association Results Viewing

**Figure 6:**
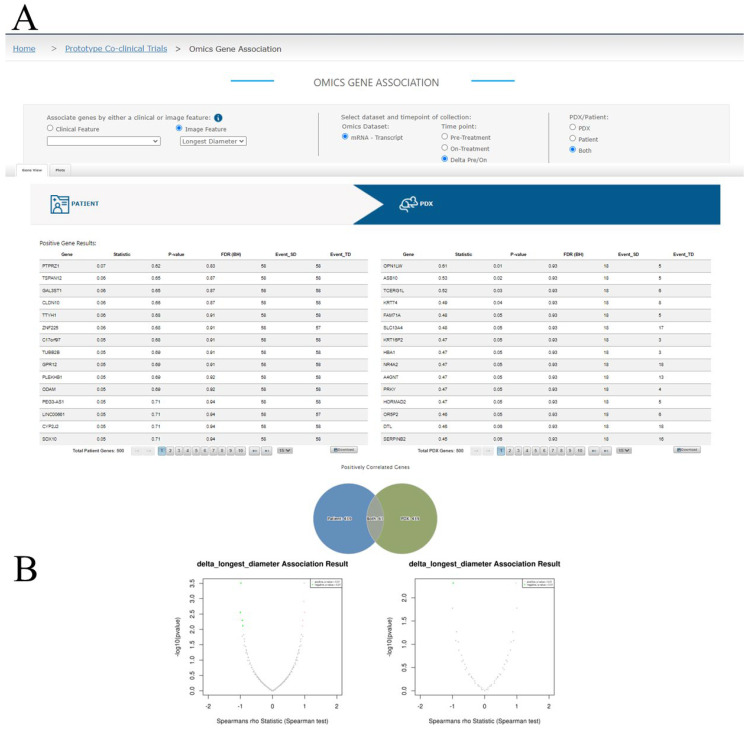
MIRACCL’s omics module. (**A**) The feature and dataset selector accepts user inputs which are passed to LinkedOmics via API for analysis. (**A**) Search results containing the top 500 and bottom 500 genes for patient and PDX are returned to MIRACCL and displayed in side-by-side tables. Venn diagrams visualizing the count of distinctive and overlapping genes between the patient and PDX cohorts are shown. (**B**) Volcano plots found on the Plots tab visually represent association results of Spearman tests for both patient and PDX cohorts.

A query interface to LinkedOmics is available in the MIRACCL portal and can be accessed from the Omics tab on the horizontal menu bar. From this query interface (Figure 6A), users can select the imaging or clinical feature of interest, along with the study time point (pre- or on-treatment, or the delta pre- vs. on- treatment) and the omics dataset to be analyzed, either patient, PDX, or both. By default, LinkedOmics currently returns the top 500 and bottom 500 genes based on the direction and strength of Spearman’s correlation, which MIRACCL renders in two data tables, one for the patient cohort and one for the PDX cohort that are displayed side-by-side (Figure 6A, only the tables for the top 500 genes are shown). Tables can be downloaded independently for further analysis. The overlaps between the top association results from patient and PDX are visualized in a Venn diagram below the tables (Figure 6A). The associations for both patient and PDX can also be visualized in two volcano plots for positive and negative associations, respectively, using the plots tab (Figure 6B).

## 4. Discussion and Future Plans for MIRACCL

### 4.1. A Focus on Breast Cancer for Prototyping MIRACCL

Breast cancer PDX models have proven to be remarkably stable during serial passage in mouse hosts over long periods of time (up to at least 20 passages) such that genetic variants, copy number alterations, and gene expression patterns (mRNA and protein) do not vary significantly, and can thus essentially be used as molecular “signatures” or “fingerprints”, characterizing each individual PDX model [13]. Related to “omic” stability over time, growth characteristics (e.g., time to progression, or uniform versus variable growth curves), and treatment responses to various agents have also proven to be remarkably stable from passage-to-passage and study-to-study [1]. This predictability allows investigators to stage studies in the most efficient manner and provides a level of confidence that results in a given agent in a given PDX that will be predictable year over year (within a range of experimental variability). This level of reproducibility is critical for longitudinal studies, and for studies conducted in multiple laboratories as PDX models become more widely disseminated from their laboratories-of-origin.

### 4.2. Opportunities and Challenges in Multi-Omics Analysis and Future Goals for MIRACCL

There are a number of omics platforms that may be used to characterize patient samples and PDX models These include whole genome DNA sequencing (WGS), whole exome DNA sequencing, assay for transposase-accessible chromatin sequencing (ATAC-Seq), chromatin structure assays (Hi-seq, CHIP-seq, etc.), single nucleotide polymorphism (SNP) arrays, mRNA transcriptomics via RNAseq or microarray technologies, mass spectrometry (MS)-based proteomics, including MS methods to detect post-translational modifications such as phosphorylation, acetylation, ubiquitinoylation, and SUMOylation, MS metabolomics (lipids, sugars, small molecules, etc.), and several others. For prototyping purposes, we chose to simulate only RNAseq data relative to treatment response. Future iterations of the platform will include more functionality for more omic data types.

Complicating matters, PDX characterization requires a separation of human and mouse analytes such that their respective contributions to the complete ome can be assessed. Several methods exist for the separation of DNA and RNA sequencing reads (e.g., Xenome [22], BBSplit https://github.com/BioInfoTools/BBMap/blob/master/sh/bbsplit.sh, Disambiguate [23] etc.), and a few for the separation of protein/peptide sequencing reads (e.g., gpGrouper) [24]. However, there are no methods that can distinguish human vs. mouse cell-derived metabolites, which could potentially compromise interpretation of experimental results.

Future plans for improving LinkedOmics interaction with MIRACCL include generation and display of gene expression heat maps and correlation plots. In addition, all correlations are currently calculated in real-time, but as additional data were added, this created a lag to display the results table in the Omics module. Consequently, future releases will include a mechanism for caching permutations of feature and gene correlations on LinkedOmics for immediate display in MIRACCL.

### 4.3. Opportunities and Challenges with Imaging and Future Plans for MIRACCL

With the MRI approach used here, there are several issues that could potentially confound interpretation. For example, the location of the tumor in a mammary fat pad makes it susceptible to motion artifacts due to its proximity to the lung, thereby limiting the accuracy of quantitative diffusion weighted MRI (DW-MRI) and dynamic contrast-enhanced MRI (DCE-MRI). The use of axial slices (as opposed to coronal or sagittal) appears to reduce these artifacts. Additionally, this location makes identification of an arterial input function for quantitative MRI difficult, yielding data that can only be analyzed with a semi-quantitative metric (e.g., the signal enhancement ratio) or by a reference region method [25].

As with omics, there are a host of quantitative imaging techniques that are readily available for application in both the pre-clinical [26] and clinical settings [27]. Beyond the usual anatomical techniques employed for RECIST analysis [28], commonly used techniques include dynamic contrast-enhanced MRI (DCE-MR), diffusion weighted MRI (DW-MRI), and fluorodeoxyglucose PET (FDG-PET). DCE-MRI acquires images before, during, and after the injection of paramagnetic contrast agent and is used to assess vessel perfusion and permeability, as well as tissue volume fractions [29]. DW-MRI yields images with and without additional magnetic field gradients that are sensitive to water motion, thereby yielding data that can report on tissue cellularity [30]. These two are frequently employed in co-clinical trials and they are the ones we utilized in the present study. Future iterations of MIRACCL will enable inclusion of these other imaging modalities.

## 5. Conclusions

Once completed, the MIRACCL platform will provide users with integrative analyses of pre-clinical PDX-based trials, clinical trials, and co-clinical trials, in which imaging features can be integrated with molecular “omic” data to address questions of interest, including treatment response assessment and prediction, as well as possible mechanisms of treatment resistance.

## Data Availability

All data are available from the corresponding author.

## Supplementary Materials

The following supporting information can be downloaded at: https://www.mdpi.com/article/10.3390/tomography9020066/s1, Figure S1: Study schema of the I-SPY2 clinical trial. Using only patient data for those with triple-negative breast cancer, baseline mRNA expression and MRI timepoints T0, T1, and T2 in the paclitaxel only (control) arm were used for MIRACCL prototyping. After random permutation of the mRNA expression values to simulate T1 expression, changes in image and omic features between T0 and T1 were calculated, and ultimate treatment response was calculated by comparison of tumor volumes at T2 (post-treatment with paclitaxel) with starting tumor volume at T0; Figure S2: (A) Display of the table of image files representing each member of a cohort. (B) Selected images displayed in ePAD for inspection and comparison; Table S1: Drug source and forumulation for PDX treatment studies.
