# Supplementary material for: Toward Practical Integration of Omic and Imaging Data in Co-Clinical Trials
Source: Tomography. 2023 Apr 10;9(2):810–28. doi: 10.3390/tomography9020066 (PMC10144684; doi:10.3390/tomography9020066)
Supplement: Supplementary file 1 [file tomography-09-00066-s001.zip › tomography-2225935-supplementary.pdf]

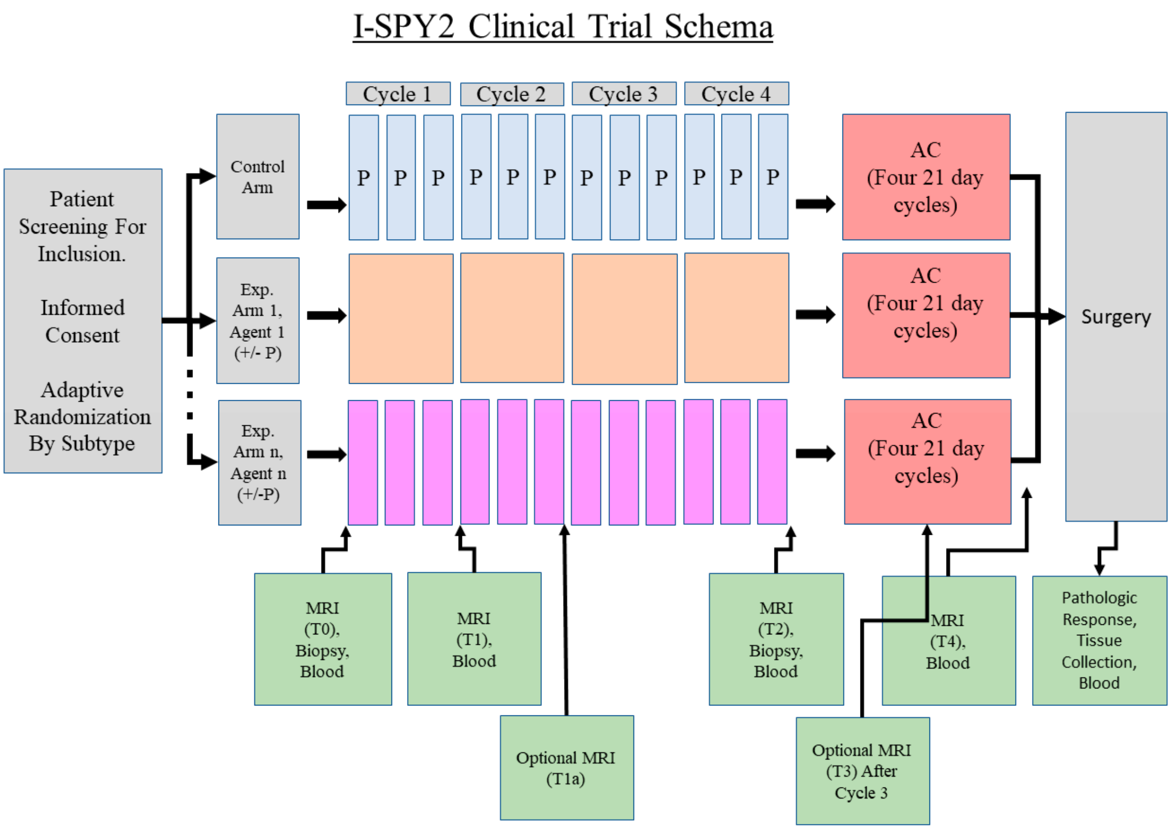

Supplemental Figure S1. Study schema of the I-SPY2 clinical trial. Using only patient data for those with triple-negative breast cancer, baseline mRNA expression and MRI timepoints T0, T1, and T2 in the paclitaxel only (control) arm were used for MIRACCL prototyping. After random permutation of the mRNA expression values to simulate T1 expression, changes in image and omic features between T0 and T1 were calculated, and ultimate treatment response was calculated by comparison of tumor volumes at T2 (post-treatment with paclitaxel) with starting tumor volume at T0.

Supplemental Table S1. Drug source and forumulation for PDX treatment studies.

Drug	Vendor	Catalog Number	Dose (mg/kg)	Concentration (mg/mL)	Vehicle	Route	Schedule
Carboplatin (Carbo)	McKesson (Teva)	740278	50	10	10 mg Mannitol per 1 mL Water	IP	Weekly
Paclitaxel (Pac)	Millipore Sigma	T7402	33	1	90% Saline/5% Kolliphor/5% Ethanol	IP	Weekly

A

COHORT
IMAGES

Total Patients showing: 39

Participant	Type	Age at Onset of Disease	Age at Baseline Study	Demographics	Pathologist Tx Response Assessment	View Image Series
BCM-15120-TG7-4412	PDX	51		White, Hispanic or Latino	pCR	Pre vs. On Treatment Images
BCM-15120-TG7-4452	PDX	51		White, Hispanic or Latino	pCR	Pre vs. On Treatment Images
BCM-15120-TG7-4465	PDX	51		White, Hispanic or Latino	pCR	Pre vs. On Treatment Images
BCM-2277-R1TG9-4825	PDX	32		Black or African American	pCR	Pre vs. On Treatment Images
BCM-2277-R1TG9-4833	PDX	32		Black or African American	pCR	Pre vs. On Treatment Images
BCM-2277-R1TG9-4840	PDX	32		Black or African American	pCR	Pre vs. On Treatment Images
BCM-3204-R3TG5-4709	PDX	42		White	no pCR	Pre vs. On Treatment Images
BCM-3204-R3TG5-4712	PDX	42		White	no pCR	Pre vs. On Treatment Images
BCM-3204-R3TG5-4721	PDX	42		White	no pCR	Pre vs. On Treatment Images
BCM-4849-R1TG15-4307	PDX	59		White, Hispanic or Latino	no pCR	Pre vs. On Treatment Images
BCM-4849-R1TG15-4309	PDX	59		White, Hispanic or Latino	no pCR	Pre vs. On Treatment Images
BCM-4849-R1TG15-4347	PDX	59		White, Hispanic or Latino	no pCR	Pre vs. On Treatment Images
BCM-BRA45-R2TG8-4503	PDX	55		Asian	no pCR	Pre vs. On Treatment Images
BCM-BRA45-R2TG8-4510	PDX	55		Asian	no pCR	Pre vs. On Treatment Images
BCM-BRA45-R2TG8-4530	PDX	55		Asian	no pCR	Pre vs. On Treatment Images
HCI-034-TG11-4204	PDX	49		White	no pCR	Pre vs. On Treatment Images
HCI-034-TG11-4205	PDX	49		White	no pCR	Pre vs. On Treatment Images
HCI-034-TG11-4223	PDX	49		White	no pCR	Pre vs. On Treatment Images
ACRIN-6698-138027	Patient	64		Asian	pCR	Pre vs. On Treatment Images

B

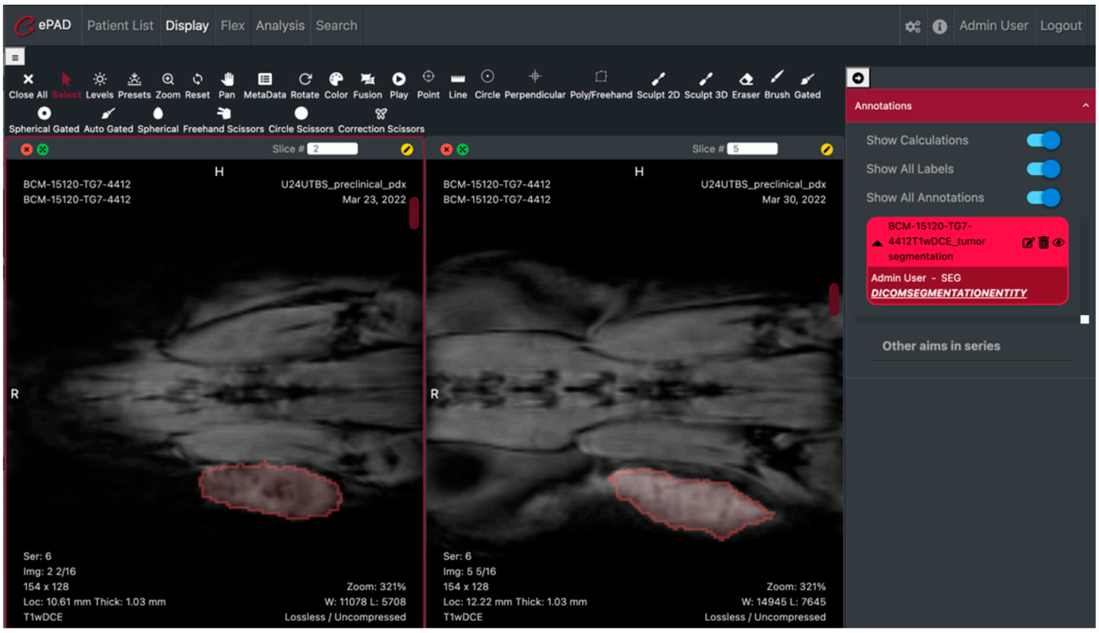

Supplemental Figure S2. (**A**) Display of the table of image files representing each member of a cohort. (**B**) Selected images displayed in ePAD for inspection and comparison.
